# *Nocardia noduli* sp. nov., a novel actinobacterium with biotechnological potential

**DOI:** 10.1007/s00203-022-02878-x

**Published:** 2022-04-13

**Authors:** Imen Nouioui, Gabriele Pötter, Marlen Jando, Michael Goodfellow

**Affiliations:** 1 Leibniz Institute DSMZ–German Collection of Microorganisms and Cell Cultures, 38124 Braunschweig, Germany; 2 School of Natural and Environmental Sciences, Newcastle University, Ridley Building 2, Newcastle upon Tyne, NE1 7RU UK

**Keywords:** Microbial systematics, Taxonomy, Actinobacteria, Novel taxa

## Abstract

A genome-based polyphasic study was undertaken to establish the taxonomic status of an actinobacterium strain isolated from an actinorhizal root nodule. Strain ncl1^T^ was found to have chemotaxonomic, cultural and morphological properties characteristic of members of the genus *Nocardia*. The strain was closely related to *Nocardia aurea* in the phylogenetic trees based on 16S rRNA gene and genome sequences. The draft genome of the strain is 8.9 Mbp in size, has a genomic DNA G + C content of 67.0% and was predicted to contain at least 19 biosynthetic gene clusters encoding for specialized metabolites. Strain ncl1^T^ was distinguished from its closest neighbour, *N. aurea* DSM 103986^T^, by a broad range of phenotypic properties and by low average nucleotide identity and digital DNA-DNA hybridization scores. Consequently, the strain represents a novel *Nocardia* species for which the name *Nocardia noduli* sp. nov. is proposed. The type strain is ncl1^T^ (CECT 30123^T^ = DSM 110878^T^). The present study provides further evidence that actinorhizal nodules are a source of novel species of *Nocardia*.

**Supplementary Information:**

The online version contains supplementary material available at 10.1007/s00203-022-02878-x.

## Introduction

Data acquired from polyphasic taxonomic and genome-based studies led to marked improvements in the classification of the genus *Nocardia* and related mycolic acid-containing bacteria (Nouioui et al. 2018) which had previously been classified using a few subjectively weighted features. The genus forms a monophyletic clade within the family *Nocardiaceae* (Goodfellow and Maldonaldo 2012) and encompasses 120 validly published species (https://lpsn.dsmz.de/genus/nocardia) most of which have been recognized using combinations of genotypic and phenotypic properties (Nouioui et al. 2020) though the steady flow of new species shows that the genus remains underspeciated (Goodfellow and Maldonaldo 2012; Nouioui et al. 2020).

*Nocardia* are well known as serious causal agents of progressive invasive infections of humans and animals, notably mycetoma and nocardiosis (Conville et al. 2018), but are also seen to be successful saprophytes as they degrade complex organic compounds (Luo et al. 2014), and as attractive sources of new drug leads (Männle et al. 2020). *Nocardia* strains have been isolated from diverse ecological settings (Goodfellow and Maldonaldo 2012), notably from soil, sediments, sponges and wastewater treatment plants (Goodfellow and Maldonado 2012), as well as from coastal marine foams (Wright et al. 2021) and healthy plant tissues (Kaewkla and Franco 2010), including nodules of actinorhizal plants (Ghodhbane-Gtari et al. 2014) thereby suggesting they may be beneficial for plant growth (Nouioui et al. 2022).

The improved classification of the genus provides an invaluable framework for the detection of new *Nocardia* species. The present study was designed to establish the taxonomic status of a *Nocardia* strain isolated from a sterilized root nodule of *Alnus glutinosa* and to gain an insight into its biotechnological potential. The isolate ncl1^T^ was the focus of a genomic-based polyphasic study which showed that it represented a new species of the genus *Nocardia* for which the name *Nocardia noduli* sp. nov. is proposed. Genome mining showed that the type strain of the species has the potential to synthesize novel natural products, notably antibiotics and anticancer compounds.

## Materials and methods

### Isolation, maintenance and cultivation

Strain ncl1^T^ was isolated from a surfaced sterilized root nodule of an *Alnus glutinosa* tree growing in Leazes Park, Newcastle upon Tyne, UK, as described by Nouioui et al. (2022). An active culture of the strain was obtained following growth on yeast extract-malt extract agar (International *Streptomyces* project [ISP] medium 2 (Shirling and Gottlieb 1966)). The strain was checked for purity and maintained in 35% (w/v) glycerol at − 80 °C, as was the type strain of *N. aurea* DSM 103986^T^*,* which was obtained from the German Collection of Microorganisms and Cell Cultures (DSMZ). Biomass for the chemotaxonomic studies carried out on the strains was harvested from ISP2 broths shaken at 200 rpm in baffled flasks for 7 days at 28 °C. The harvested cells were washed in distilled water and freeze dried.

### Cultural and morphological properties of strain ncl1^T^

Acid-alcohol-fast staining was carried out following growth on ISP2 agar for 10 days at 28 °C using the Ziehl–Neelsen method (Gordon 1967). Hanging drop preparations were examined to check for motility. The micromorphological properties of the strain were observed by light microscopy of the Gram stained smear. The ability of the strain to grow at a range of temperatures (4 °C, 10 °C, 15 °C, 25 °C, 28 °C, 37 °C, 42 °C and 45 °C) and pH (pH4.0–9.0 at intervals of 0.50 unit) was recorded from ISP2 agar plates. The pH levels were achieved using KH_2_PO_4_/HCl, KH_2_PO_4_/K_2_HPO_4_ and K_2_HPO_4_/NaOH buffer systems. Growth and cultural properties were determined after incubation for 7 days at 28 °C on GYM agar (DSMZ medium 65), nutrient agar (NA, (MacFaddin 1986)), peptone-meat extract-glucose agar (DSMZ medium 250), tryptic soy agar (TSA, (MacFaddin 1986)) and ISP media 2, 3, 4 (Shirling and Gottlieb 1966). The ability of the strain to grow under anaerobic conditions was determined by inoculating it onto ISP2 agar and incubating at 28 °C for 7 days using anaerobic bag system (Sigma-Aldrich 68,061).

### Molecular identification and genome sequencing

DNA was extracted from strain ncl1^T^ prior to PCR-mediated amplification of a 16S rRNA gene (Weisburg et al. 1991) and sequencing using the Sanger method (Sanger and Coulson 1975; Sanger et al. 1977). Genomic DNA of the strain was extracted, purified and quantified following the protocol provided by MicrobesNG (Birmingham, UK, https://microbesng.com/microbesng-faq/), then sequenced on an Illumina HiSeq 250 next generation sequencing platform using the 250 bp paired end sequencing protocol at MicrobesNG. Assembly of the reads and associated procedures were also carried out at MicrobesNG. The draft genome sequence of the strain was annotated using the RAST-SEED webserver (Aziz et al. 2008). The completeness of the genome sequence of the strain was estimated using BUSCO v.5.1.2 (Seppey et al. 2019).

### Genome comparison

The draft genome of strain ncl1^T^ was compared with that of *N. aurea* SYSU K10002^T^ (accession number NZ_QCYJ00000000.1) for several genomic features, notably genome size and digital DNA G + C content. Average nucleotide identity (ortho ANI; Lee et al. 2016), average amino acid identity (AAI) (Konstantinidis and Tiedje 2005) and digital DNA-DNA hybridization (dDDH) (Meier-Kolthoff et al. 2013) similarities were determined from the draft genomes using the ANI calculators from the EzBioCloud (https://www.ezbiocloud.net/tools/ani) and the Genome-to-Genome Distance Calculator GGDC 2.1 (http://ggdc.dsmz.de) webserver, respectively.

### Phylogeny

An almost full length 16S rRNA gene sequence extracted directly from the draft genome of strain ncl1^T^ (1523 pb) was deposited in the GenBank under accession number OK597210. This sequence was identical to the 16S rRNA gene sequence obtained using the Sanger method. The gene sequence was compared with corresponding gene sequences of the type strains of closely related *Nocardia* species retrieved from the EzBioCloud database (Yoon et al. 2017). 16S rRNA gene and whole-genome phylogenetic trees were inferred using the Type Strain Genome Server (TYG), available on the GGDC webpage (Meier-Kolthoff and Göker 2019).

### Phenotypic and chemotaxonomic properties

Strain ncl1^T^ and *N. aurea* DSMZ 103986^T^ were examined for a range of phenotypic features; notably for their ability to use sole carbon and nitrogen sources; to grow at various pH values and in the presence of antibiotics and inorganic inhibitory compounds using GENIII microplates and an Omnilog device (Biolog Inc., Hayward, CA, USA), as described by Nouioui et al. (2020). The resultant data were analysed using opm package version 1.3.36 (Vaas et al. 2013). Enzymatic profiles of the strains were established using API-ZYM kits (BioMerieux, Lyon, France) by following the manufacturer’s instructions. Catalase and oxidase tests were determined using standard procedures. All of the tests cited above were performed in duplicate.

Standard chromatographic procedures were used to establish the polar lipid profile and diaminopimelic acid isomers using the integrated method of Minnikin et al. (1984) and the protocol of Schleifer and Kandler (1972), respectively. Whole-organism sugar patterns were determined after Lechevalier and Lechevalier (1970) and Staneck and Roberts (1974). Mycolic acids were extracted and purified as described by Goodfellow et al. (1976) and identified by gas chromatography (Agilent Technologies 6890 N, Santa Clara, CA, USA).

Cellular fatty acids extracted from the strain and *N. aurea* DSM 103986^T^ were methylated following Miller (1982), as modified by Kuykendall et al. (1988) and analysed by gas chromatography using the Agilent instrument. The resultant peaks were identified using the standard Microbial Identification (MIDI) system version 4.5 and the ACTIN6 database (Sasser 1990).

### Biosynthetic gene clusters coding for specialized metabolites

The presence of natural product-biosynthetic gene clusters (NP-BGCs) in the genome of the isolate and the *N. aurea* strain were detected using antiSMASH, version 6.0 (Blin et al. 2019) with the detection relaxed option.

## Results and discussion

### Cultural, chemotaxonomic and biochemical properties

Strain ncl1^T^ did not grow under anaerobic conditions, but did grow from 28 °C to 37 °C, optimally at 28 °C, and from pH 6–7.5. It formed orange colonies on GYM and ISP2 media and orange yellowish aerial hyphae on DSM 250 medium. It was also found to be catalase positive and oxidase negative.

Excellent congruence was found between the phenotypic data acquired on the duplicated tests. Table 1 shows that the isolate and *N. aurea* DSM 103986^T^ can be distinguished using a combination of phenotypic features though they also have many properties in common. Only the isolate was able to metabolise d-arabitol, D-fucose, *β*-gentiobiose, glucuronamide, *N*-acetyl-D-glucosamine, d-mannitol, methyl pyruvate and l-rhamnose (sugars), gelatin (polymer), and was resistant to vancomycin. In contrast, *N. aurea*, unlike the strain, metabolised 3-*O*-methyl-d-glucose, d-turanose, *β*-methyl-d-glucoside, and d-salicin (sugars) and was resistant to fusidic acid and minocycline.

**Table 1:** Phenotypic data that distinguish the isolate from the type strain of *N. aurea,* its closest phylogenomic neighbour

Features	Isolate ncl1^T^	*N. aurea* DSM 103986^ T^
GEN III Biolog microplate tests		
Sugars		
D-Arabitol, D-fucose, β-gentiobiose, glucuronamide, *N*-acetyl-D-glucosamine, D-mannitol, methyl pyruvate and L-rhamnose,	+	−
D-Fructose, D-mannose, sucrose	+	w
3-*O*-methyl-D-Glucose, β-methyl-D-glucoside, D-salicin and D-turanose	−	+
Oxidation of amino acids		
L-Alanine, L-arginine, D-aspartic acid and glycine-proline	+	−
Organic acids		
α-*keto*-butyric acid, citric acid, L-glutamic acid, α-*keto*-glutaric acid, L-lactic acid, D-lactic acid methyl ester, and L-pyroglutamic acid	+	−
Acetoacetic acid, γ-amino-n-butyric acid, α-hydroxy-butyric acid, β-hydroxy-butyric acid, L-galactonic acid-γ-lactone, D-gluconic acid, quinic acid and D-saccharic acid	+	w
Polymer		
Gelatin	+	−
Tolerance to		
Lithium chloride and sodium formate	w	+
Resistant to		
Fusidic acid and minocycline	−	+
Vancomycin	+	−
Aztreonam	w	+

Both strains were able to reduce D-fructose-6-phosphate, D-galactose, D-glucose, D-glucose-6-phosphate, L-fucose, glycerol, *myo*-inositol, *n*-acetyl-β-D-mannosamine, D-raffinose (sugars); acetic acid, bromo-succinic acid, butyric acid, D-galacturonic acid, D-glucuronic acid, D-malic acid, L-malic acid, mucic acid, propionic acid, (organic acids); L-aspartic acid, L-histidine, D-serine #1 and 2, L-serine (amino acids); inosine (nucleoside); and grow in the presence of 4% NaCl, 1% sodium lactate, sodium bromate (salts); guanidine hydrochloride, niaproof, potassium tellurite, tetrazolium blue, tetrazolium violet, Tween 40 (inhibitory compounds); were resistant to lincomycin, nalidixic acid, rifamycin SV, troleandomycin (antibiotics); and grew at pH5.0–6.0; and were positive for α-galactosidase, valine aminopeptidase, β-glucosidase, n-acetyl-β-glucosaminidase, naphthol-AS-BI-phosphohydrolase (API ZYM). Neither strain was able to metabolise D-cellobiose, dextrin, D-maltose, D-melibiose, D-sorbitol, D-trehalose, *n*-acetyl-D-galactosamine, stachyose, α-D-lactose (sugars); *n*-acetyl-neuraminic acid, *p*-hydroxy-phenylacetic acid (organic acids); pectin (polymer); to grow in the presence of 8% NaCl; to produce alkaline phosphatase, leucine aminopeptidase, cystine aminopeptidase, chymotrypsin, acid phosphatase, β-galactosidase, α-glucosidase, β-glucuronidase, α-fucosidase trypsin, and α-mannosidase (API ZYM). Both strains showed a weak reaction for esterase (C4), esterase lipase (C8), lipase (C14) (API ZYM)

Symbols: − negative reaction; *w* weak reaction; + , positive reaction

Chemotaxonomic, colonial and micromorphological features of the strain were consistent with its classification in the genus *Nocardia* (Goodfellow and Maldonaldo 2012). The strain was found to be aerobic, nonmotile, Gram-stain-positive, partial acid-alcohol-fast and formed substrate and aerial hyphae that fragmented into rod and coccoid-like elements. It was shown to have *meso*-A2pm as the wall peptidoglycan, arabinose, galactose and glucose as whole-organism sugars; mycolic acids with 46–64 carbon atoms and a polar lipid profile composed of diphosphatidylglycerol, phosphatidylethanolamine (diagnostic phospholipid) and phosphatidylinositol and spots corresponding to unknown lipid, phosphoglycolipid and glycolipid components (Figure S1). The strain and the *N. aurea* strain were found to be rich in straight chain, saturated, unsaturated and 10-methyl (tuberculostearic) fatty acids though quantitative differences were found between the various components (Table S1). Both strains feature *meso*-A2pm as the wall peptidoglycan and arabinose, galactose and glucose as diagnostic sugars, but can be distinguished as the strain also contain glucose and the *N. aurea* strain ribose (Fang et al. 2019).

### Genome comparison

The draft genome sequence of the strain has been deposited in the GenBank database under accession number JAFMPL000000000. The genome of the strain has a BUSCO score of 99.7% complete genes (355/356 from the actinobacteria_class_odb10). The genome sizes of strain ncl1^T^ and the type strain of *N. aurea* were 8.9 Mbp and 8.3 Mbp, respectively, and the corresponding genomic DNA G + C contents were 67.0 and 67.4%. The dDDH similarity between the strains was 61.7% (Table 2), a value much lower than the corresponding threshold of 70% used to assign closely related strains to the same species (Wayne et al. 1987). However, the ANI value between these strains was shown to be 95%, and the AAI 93.7%, which is below the cut-off point of 95–96% for species demarcation (Goris et al. 2007; Richter and Rosselló-Móra 2009; Thompson et al. 2013).

**Table 2:** Digital DNA-DNA hybridization score between the genome sequence of isolate ncl1^T^ and its closest phylogenomic neighbours

Reference type strains	dDDH (%)
*Nocardia aurea* SYSU K10002^T^	61.7
*Nocardia arizonensis* NBRC 108935^T^	24.1
*Nocardia takedensis* NBRC 100417^T^	23.5
*Nocardia lijiangensis* NBRC 108240^T^	23.2
*Nocardia bhagyanarayanae* DSM 103495^T^	23.1
*Nocardia xishanensis* NBRC 101358^T^	23.1
*Nocardia vulneris* NBRC 108936^T^	22.5
*Nocardia tenerifensis* DSM 44704^T^	22.5
*Nocardia asiatica* NBRC 100129^T^	22.5
*Nocardia brasiliensis* NBRC 14402^T^	22.5
*Nocardia exalbida* NBRC 100660^T^	22.4
*Nocardia farcinica* DSM 43257^T^	22.4
*Nocardia higoensis* NBRC 100133^T^	22.1
*Nocardia altamirensis* NBRC 108246^T^	22.1
*Nocardia asteroides* DSM 43373^T^	21.5

### Phylogeny

**Fig. 1:**
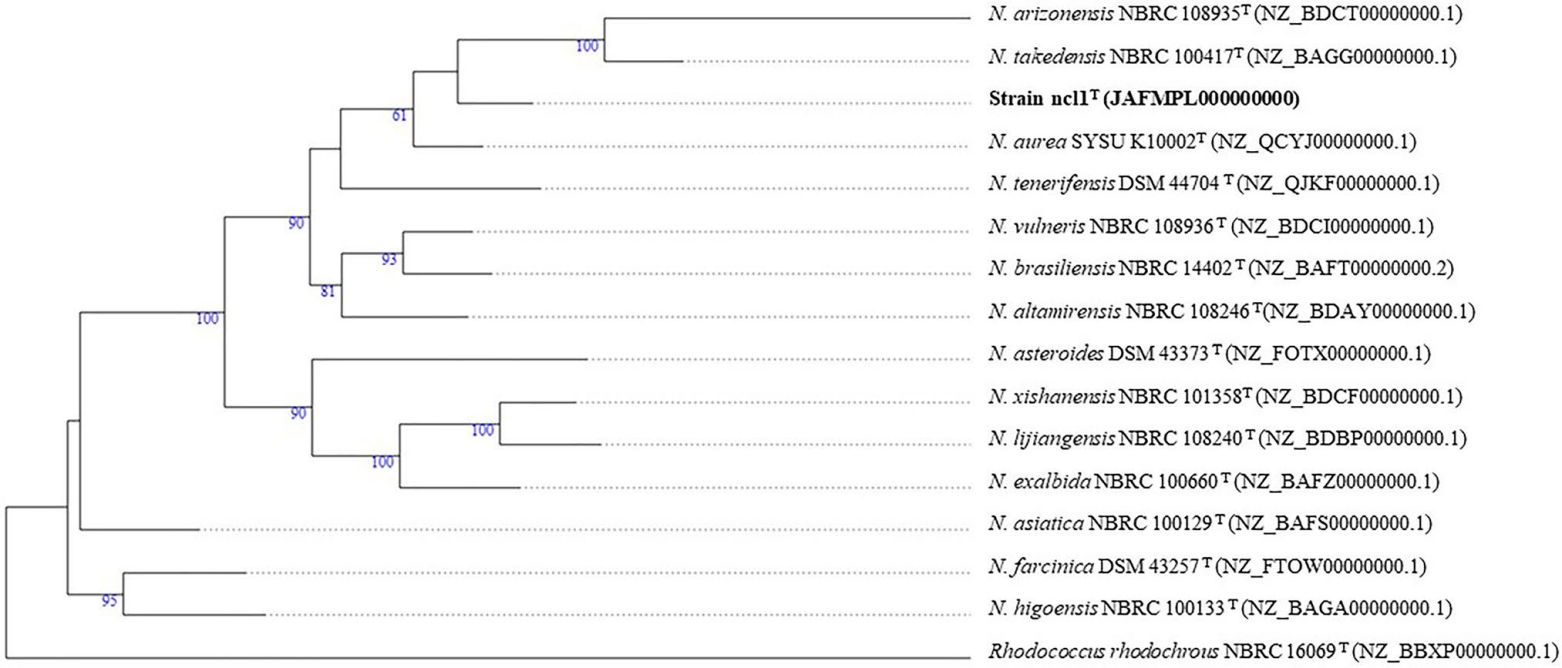
Maximum likelihood phylogenetic tree based on 16S rRNA gene sequences, showing the relationships between strain ncl1^T^ and closely related *Nocardia* type strains. The numbers above branches are bootstrap support values > 60%

**Fig. 2:**
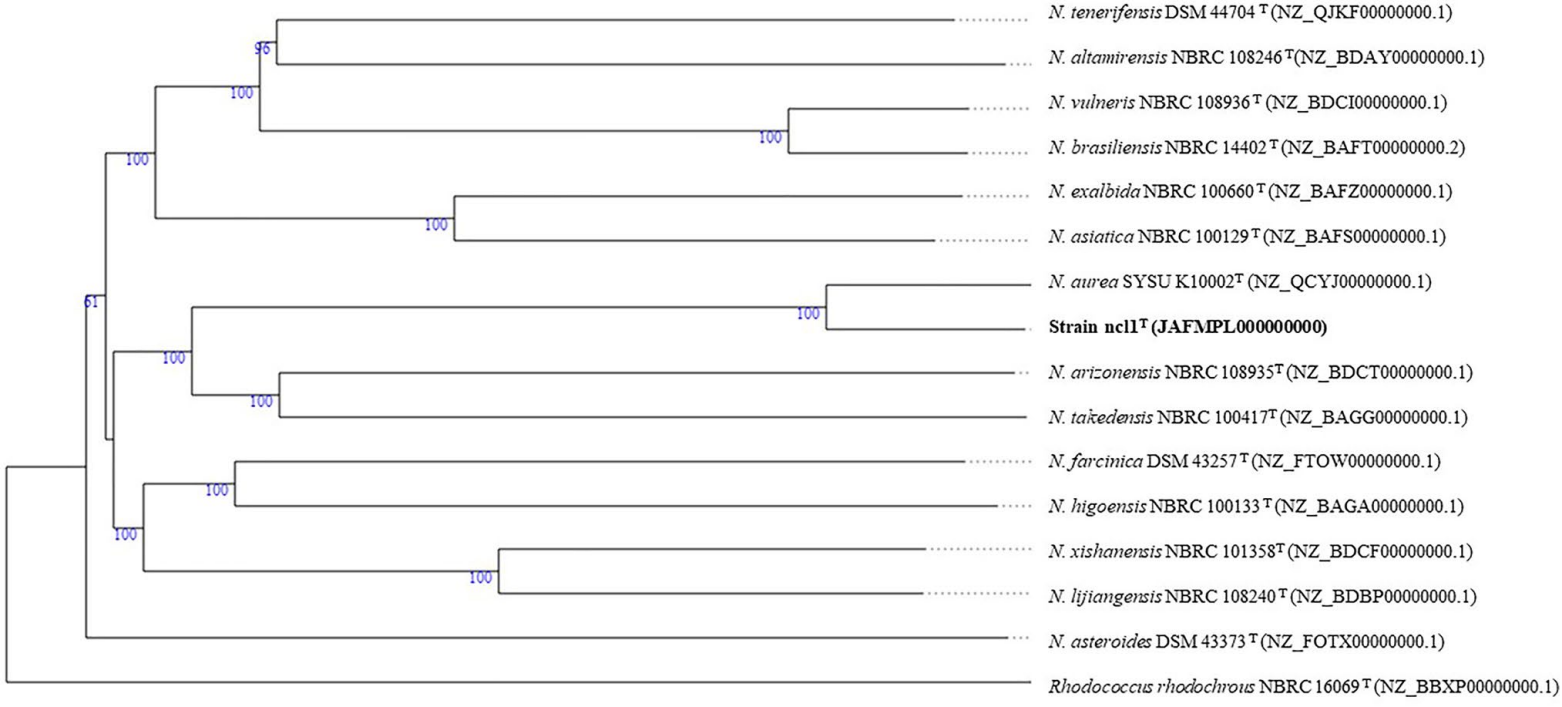
Phylogenomic tree showing relationships between strain ncl1^T^ with its closest phylogenomic relatives. The numbers above branches are GBDP pseudo-bootstrap support values from 100 replications

The maximum-likelihood 16S rRNA *Nocardia* tree (Fig. 1) showed that the strain formed a well separated branch in a clade that included the type strains of seven *Nocardia* species. Its closest relative was found to be *N. aurea* SYSU K10002^T^ (99.4%) in the phylogenomic tree. Greater confidence can be placed in the topology of phylogenomic trees as they are generated from millions of unit characters not limited to about 1500 as in the case of corresponding 16S rRNA gene trees (Nouioui et al. 2018). It is evident from the phylogenomic tree that the strain and *N. aurea* SYSU K10002^T^ formed a well-supported subclade that is most closely related to a second well delineated taxon composed of *Nocardia arizonensis* NBRC 108935^T^ and *Nocardia takedensis* NBRC 100417^T^ (Fig. 2). The resultant well-supported clade was seen to be distant from corresponding clades encompassing several *Nocardia* species, notably *Nocardia brasiliensis* NBRC 14402^T^ and *Nocardia vulneris* NBRC 108936^T^, and *N. altamirensis* NBRC 108246^T^ and *Nocardia tenerifensis* DSM 44704^T^, respectively.

### Biosynthetic gene clusters coding for specialized metabolites

AntiSMASH predicts NP-BGCs and potential products based on the percentage of genes from the closest known bioclusters which show BLAST hits in the genomes of strains of interest. In the present study, the genome of strain ncl1^T^ and the *N. aurea* DSM 103986^T^ contained 19 and 31 BGCs, respectively. Many of the bioclusters were predicted to encode for different classes of specialized metabolites, notably non-ribosomal peptide synthases (NRPS); type 1 and 2 polyketide synthases (PKS) and terpenes (Table 3), thereby providing further evidence that nocardiae have the capacity to produce diverse natural products (Männle et al. 2020). The genome of each strain contained bioclusters associated with the synthesis of nocobactin (87% gene identity), a UV-active siderophore first reported from cultures of *Nocardia asteroides* (Ratledge and Snow 1974), and streptobactin (11% gene identity), a tricatechol-type siderophore from a marine-derived *Streptomyces* strain which showed iron-chelating activity (Matsuo et al. 2011). The strains were also found to have bioclusters predicted to encode for the volatile terpenes geosmin (100% gene identity), isorenieratene (57% gene identity), 2-methylisoborneol (75% gene similarity), and acyldepsipeptide (10% gene identity). The latter was isolated from a marine-derived *Streptomyces atratus* strain and was found to be active against *Mycobacterium tuberculosis* (Sun et al. 2019). The strains were found to have the capacity to produce caniferolides A to D with 12% to 14% gene identity, respectively. The latter are PKS compounds which were initially detected in *Streptomyces caniferus* and shown to inhibit the growth of *Aspergillus* spp. and *Candida albicans* (Pérez-Victoria et al. 2019); caniferolide A has been used to treat Alzheimer’s disease (Alvariño et al. 2019).

**Table 3:** Biosynthetic gene clusters detected in the genome sequences of isolate ncl1^T^ and *N. aurea* SYSU K10002^T^ its closest phylogenomic neighbour

Type	Closest known cluster	Isolate ncl1^T^	*N. aurea* SYSU K10002^T^
		Gene sequence similarity (%)	
NRPS	Cosmomycin D	–	5
NRPS	Pepticinnamin E	–	6
NRPS	Acyldepsipeptide 1	–	10
T1PKS, NRPS, siderophore	Ficellomycin	–	3
T1PKS, NRPS	Abyssomicin C / *atrop*-abyssomicin C	–	25
T1PKS	Tetarimycin A / tetarimycin B	–	8
T2PKS	Olimycin A / olimycin B	–	29
NRP	Atratumycin	5	-
NRP	Acyldepsipeptide 1	10	10
NRP	Streptobactin	11	11
NRP	Vazabitide A	10	10
NRP + Polyketide	Polyoxypeptin	8	-
NRP + Polyketide	Nocobactin NA	87	87
Polyketide: Modular type I	Argimycin PI / argimycin PII / nigrifactin / argimycin PIV / argimycin PV / argimycin PVI / argimycin PIX	10	-
Polyketide: Modular type I	Caniferolide A / caniferolide B / caniferolide C / caniferolide D	12	14
Polyketide: Type II	Cinerubin B	14	–
Polyketide	Cyphomycin	2	–
Polyketide	BE-7585A	23	23
RiPP: Bottromycin	Bottromycin A2	12	12
RiPP-like, NRPS	Funisamine	–	7
RiPP: Thiopeptide	GE2270	9	–
Ranthipeptide	Calicheamicin	4	2
Terpene	Geosmin	100	100
Terpene	Isorenieratene	57	57
Terpene	2-Methylisoborneol	75	75
Ectoine	Ectoine	–	100
NAPAA	Phosphonoglycans	–	3
Betalactone	Platencin	–	6
Saccharide + Polyketide: Modular type I + Polyketide: Type II + Other: Aminocoumarin	Simocyclinone D8	34	–
hglE-KS	Xantholipin	–	6

– not detected

Many of the remaining bioclusters found in the genomes of the two strains were found to be strain specific (Table 3). Strain ncl1^T^, for instance, contained BGCs predicted to encode for atratumycin (5% gene identity), cinerubin B (14% gene identity), an anthracycline antibiotic produced by a *Streptomyces* strain which has antitumor properties (Silva et al. 2020); cyphomycin (2% gene identity), a PKS from a marine-derived *Streptomyces* strain which is used to control multi-drug resistant fungal pathogens (Chevrette et al. 2019); polyoxypeptin A, which exhibits potent apoptosis-inducing activity towards human pancreatic carcinomic cells (Du et al. 2014), and simocyclinone D8, a cytostatic angucyclinone antibiotic from a *Streptomyces* strain which is active against Gram-stain-positive bacteria and tumour cell lines (Schimana et al. 2000).

In contrast, only the *N. aurea* strain has bioclusters associated with the synthesis of cosmomycin D (5% gene identity), an anthracycline antibiotic which shows antitumor activity (Ando et al. 1985); platencin (6% gene identity), a betalactone which inhibits multi-drug resistant *M. tuberculosis* strains (Martens and Demain 2011); pepticinnamin E (6% gene identity), a farnesyl transferase inhibitor used in cancer, malaria, and trypanosome therapy (Santa et al. 2019); xantholipin, a polycyclic xanthone antibiotic derived from *Streptomyces flavogriseus*, which was found to be cytotoxic and to show antibacterial activity against Gram-positive bacteria, including methicillin-resistant *Staphylococcus aureus* and vancomycin-resistant *Enterococcus faecalis* (Wu et al. 2017). In addition, the aziridine alkaloid antibiotic ficellomycin (3% gene identity) and the type I PKs compounds tetarimycin A and B (3% gene identity) inhibit multi-drug resistant *S. aureus* strains (Kallifidas et al. 2012; He et al. 2018). The *N. aurea* strain also has the capacity to produce abyssomicin C/ *atrop*-abyssomicin C (25% gene similarity), polycyclic macrolactones, first detected in the type strain of *Verrucosispora maris* (now *Micromonospora maris*) (Nouioui et al. 2018), which show pronounced activity against multi-drug resistant Gram-positive bacteria (Fiedler 2021). Additional compounds associated with each of the strains are shown in Table 3.

It is evident from the results outlined above that strain ncl1^T^ and *N. aurea* DSM 103986^T^, its closest phylogenomic neighbour, have genomes rich in BGCs predicted to encode for novel polyketides. These results provide further evidence that nocardiae should feature more prominently in natural product discovery campaigns designed to find novel antibiotics of therapeutic value. This prospectus is in line with the view that novel *Nocardia* species represent a prolific source of natural products, one that rivals that of better characterised genera such as *Amycolatopsis* and *Streptomyces* (Männle et al. 2020).

## Conclusions

Nodular tissues provide shelter and a plentiful supply of carbohydrates for bacteria, including actinobacteria found to belong to the genera *Frankia*, *Micromonospora* and *Streptomyces*. The present study provides further evidence that novel *Nocardia* species are associated with actinorhizal nodules though ecophysiological studies are needed to establish their ecological role and potential as inoculants to promote plant growth. Further, their ability to inhibit phytopathogens offers a role as an eco-friendly alternative to the use of pesticides in agriculture.

It can be concluded from the phylogenetic and phylogenomic trees that the strain forms a distinct lineage that is most closely related to the type strain of *N. aurea*. However, it can be distinguished from the latter using a broad range of phenotypic data and by low ANI and dDDH similarities. It is, therefore, proposed that strain ncl1^T^ represents a new species within the genus *Nocardia* for which the name *Nocardia noduli* sp. nov. is proposed.

## Description of *Nocardia noduli*. sp. nov

*Nocardia noduli* (no’du.li. L. gen. dim. n. *noduli*, of a nodule, referring to the isolation of the type strain from a root nodule of *Alnus glutinosa*).

Aerobic, Gram-stain-positive, non-motile organism which forms substrate and aerial hyphae that fragment into rod-and coccoid-like elements. Orange colonies are formed on GYM and ISP2 agar. Grows from pH 5–7.5, optimally at pH 7.0 and from 28 to 37 °C, optimally at 28 °C, and in presence of up to 4% w/v sodium chloride. It is catalase positive, oxidase negative, able to metabolise D-arabitol, D-fucose, β-gentiobiose, glucuronamide, *N*-acetyl-d-glucosamine, D-mannitol, methyl pyruvate and L-rhamnose (sugars), gelatin (polymer), is resistant to vancomycin and positive for α-galactosidase, valine aminopeptidase, β-glucosidase, n-acetyl-β-glucosaminidase and naphthol-AS-BI phosphohydrolase. The diamino acid of the peptidoglycan is *meso*-A2pm, the whole-organism sugars are arabinose, galactose and glucose; the predominant fatty acids (> 10%) are C_16:0_, C_18:1_ ω9c and 10-methyl C_18:0_, and the mycolic acids have 46–64 carbon atoms. The polar lipids consist of diphosphatidylglycerol, phosphatidylethanolamine, phosphatidylinositol, a glycophospholipid and two unidentified components. The genome size of the strain is 8.9 Mbp and its genomic DNA G + C content 67.0%. The genome is rich in biosynthetic gene clusters predicted to encode for specialized metabolites, notably antibiotics.

The type strain, ncl1^T^ (CECT 30123^T^ = DSM 110878^T^), was isolated from the root nodule of an *Alnus glutinosa* plant growing in Leazes Park, Newcastle upon Tyne, UK.

The 16S rRNA gene and genome sequences have been deposited in the DDBJ/ENA/GenBank databases under the accession numbers OK597210 and JAFMPL000000000, respectively.

The Whole Genome Shotgun project has been deposited at DDBJ/ENA/GenBank under the accession number JAFMPL000000000. The version described in this paper is version JAFMPL010000000.

## Supplementary Information

Below is the link to the electronic supplementary material.

Supplementary file1 (DOCX 140 KB)
