# Supplementary material for: Nocardia noduli sp. nov., a novel actinobacterium with biotechnological potential
Source: Arch Microbiol. 2022 Apr 13;204(5):260. doi: 10.1007/s00203-022-02878-x (PMC9007811; doi:10.1007/s00203-022-02878-x)
Supplement: Supplementary file 1 — Supplementary file1 (DOCX 140 KB) [file 203_2022_2878_MOESM1_ESM.docx]

***Nocardia noduli* sp. nov., a novel actinobacterium with biotechnological potential**

Imen Nouioui^1*^, Gabriele Pötter^1^, Marlen Jando^1^, Michael Goodfellow^2^

^1^Leibniz Institute DSMZ–German Collection of Microorganisms and Cell Cultures, 38124 Braunschweig, Germany

^2^School of Environmental Sciences, Newcastle University, Ridley Building 2, Newcastle upon Tyne, NE1 7RU, United Kingdom

Corresponding author : Imen Nouioui, imen.nouioui@dsmz.de

**Figure S1.** Two-dimensional TLC plates of polar lipids extracted from isolate ncl1^T^ stained with molybdatophosphoric acid (SigmaP1518). Key: DPG, diphosphatidylglycerol; PE, phosphatidylethanolamine; PI, phosphatidylinositol; PGL, phosphoglycolipid; GL, glycolipid, L, lipid. Solvent1: chloroform: methanol: distilled water (65:25:4 v/v/v/); solvent 2: chloroform: glacial acetic acid: methanol: distilled water (80:12:15:4 v/v/v).

**Table S1.** Fatty acid profiles of isolate ncl1^T^ and *N. aurea* DSM 103986^T^, its phylogenomic neighbour

| Fatty acids | Isolate ncl1^T^ (%) | *N. aurea* DSM DSM 103986^T^ |
| --- | --- | --- |
| C_16: 0_ | 36.3 | 31.4 |
| C_18:0_ ω 9c | 24.9 | 15.2 |
| C_18:0_ | 7.7 | 6.4 |
| C_18:0_ 10-methyl (tuberculostearic acid) | 13.7 | 24.2 |
| C_20:1_ ω 9c | 4.8 | 7.7 |
| Sum feature 3 | 6.9 | 11.7 |

Only fatty acid features with a percentage above 5% are displayed. Sum feature 3; C_16:1_ ω 7c/15 iso 2OH.
